# Enhancing Guideline-Directed Medical Therapy at Discharge Through Collaborative Care: Evidence from the TIME-HF Cluster Randomized Controlled Trial

**DOI:** 10.12688/wellcomeopenres.26369.1

**Published:** 2026-05-20

**Authors:** Panniyammakal Jeemon, Athira Thottathan, Mariyam Alex, Sunaib Ismail, Reshmy Mohan, Safvan Karkuzhiyil, Bahuleyan Charantharalyil Gopalan, Chandgalu Javaregowda Devaraju, Eapen Punnoose, Gopalan Rajendiran, Govindan Unni, Jabir Abdullakutty, Jayakumar Balakrishnan, Joyal J Kandathil, Johny Joseph, Justin Paul Gnanaraj, Madhu Sreedharan, Meera R Pillai, Mahesh Nalin Kumar, Paul Thomas, Placid Sebastian, Rachel Daniel, Rajeev Edakutty, Sajan Ahmad, Salman Salahuddin, Shafeeq Mattummal, Stigi Joseph, Sunil Pisharody, Natesan Syam, Tiny Nair, Veena Nanjappa, Vijayan Ganesan, Vijo George, Anu Mary Chacko, Sanjay Ganapathi, Sivadasanpillai Harikrishnan

**Affiliations:** 1 Sree Chitra Tirunal Institute for Medical Sciences and Technology, Thiruvananthapuram, Kerala, India; 2 Ananthapuri Hospitals and Research Institute, Thiruvananthapuram, Kerala, India; 3 Sri Jayadeva Institute of Cardiovascular Sciences and Research, Bengaluru, Karnataka, India; 4 Malankara Orthodox Syrian Church Medical College and Hospital, Kolenchery, Kerala, India; 5 PSG Institute of Medical Sciences and Research, Coimbatore, Tamil Nadu, India; 6 Jubilee Mission Medical College and Research Institute, Thrissur, Kerala, India; 7 Lisie Hospital, Kochi, Kerala, India; 8 Thangam Hospital of PMRC, Palakkad, Kerala, India; 9 St. Gregorios Memorial Mission Hospital, Parumala, Kerala, India; 10 Caritas Hospital, Kottayam, Kerala, India; 11 Madras Medical College and Government General Hospital, Rajiv Gandhi Government General Hospital, Chennai, Tamil Nadu, India; 12 NIMS Hospital, Thiruvananthapuram, Kerala, India; 13 KIMSHEALTH, Thiruvananthapuram, Kerala, India; 14 General Hospital, Ernakulam, Kerala, India; 15 Aster MIMS Hospital, Kannur, Kerala, India; 16 NS Memorial Institute of Medical Sciences, Kollam, Kerala, India; 17 District Cooperative Hospital, Calicut, Kerala, India; 18 Aster MIMS Hospital, Calicut, Kerala, India; 19 Little Flower Hospital and Research Centre, Angamaly, Kerala, India; 20 EMS Memorial Cooperative Hospital and Research Centre Ltd, Malappuram, Kerala, India; 21 Government District Hospital, Kollam, Kerala, India; 22 PRS Hospital, Thiruvananthapuram, Kerala, India; 23 National Institute of Technology Calicut, Kozhikode, Kerala, India

**Keywords:** Heart failure with reduced ejection fraction; HFrEF, Guideline-directed medical therapy, GDMT, Collaborative Care Model

## Abstract

**Background:**

Nurse-led care supported by mobile health (mHealth) technologies offers a feasible strategy to strengthen heart failure (HF) management in resource-constrained settings. This study evaluated the impact of an mHealth-enabled, nurse-led collaborative care model on prescription of guideline-directed medical therapy (GDMT) at discharge among patients with heart failure with reduced ejection fraction (HFrEF) in India.

**Methods:**

The TIME-HF study was a multicenter, open-label, cluster-randomized controlled trial conducted across 22 centers enrolling patients with HFrEF. Centers were randomized 1:1 to an intervention arm (11 centers; 755 patients) or enhanced usual care (11 centers; 752 patients). In the intervention arm, nurses used an mHealth application to review GDMT prescriptions and recommend treatment modifications to physicians. GDMT at discharge was assessed individually and using a composite score. Intervention effects were evaluated using multivariable mixed-effects logistic regression models.

**Results:**

Participants were predominantly male (77.6%) with a mean age of 61.9 years (SD 12.1). Hypertension (56.1%), diabetes (62%), and chronic kidney disease (18.4%) were common comorbidities. Ischemic heart disease was the leading etiology (77.4%), and 48.8% were in NYHA class III/IV. Baseline characteristics were similar between groups. At discharge, 20% received all four GDMT components, with higher use in the intervention group than usual care (27% vs 13%, p < 0.001; adjusted OR 1.61, 95% CI 1.48–1.75). Intervention participants were also more likely to achieve a high GDMT score (OR 1.13, 95% CI 1.04–1.22).

**Conclusion:**

An mHealth-supported nurse-led discharge medication review improved GDMT use in HFrEF and may help reduce preventable HF mortality in India.

**Trial registration: Clinical Trial Registry of India (CTRI/2021/11/037797), Registered on 03/11/2021; the study is ongoing.**

## Plain Language summary

Heart failure with reduced ejection fraction is a serious condition where the heart cannot pump blood effectively. Proven treatments, known as guideline-directed medical therapy (GDMT), can reduce deaths and hospital visits, but many patients do not receive all recommended medicines, especially in low-resource settings like India.

We conducted the TIME-HF study to test whether a nurse-led care model supported by a mobile health (mHealth) tool could improve prescribing of these treatments at hospital discharge. This study took place across 22 hospitals in India and included 1,507 patients. Hospitals were randomly assigned to either usual care or an intervention where nurses used a mobile app to review medications and suggest improvements to doctors.

We found that patients in the intervention group were more likely to receive all recommended medicines at discharge (27% compared with 13% in usual care). Overall treatment scores were also slightly higher. However, only one in five patients received all recommended therapies.

This study shows that structured, nurse-led, digitally supported care can improve early treatment. However, longer-term strategies are still needed to address follow-up care, medication costs, and adherence to achieve lasting benefits.

## Introduction

Heart failure (HF) is a growing global public health challenge, affecting approximately 64 million people worldwide. ^1^ It contributes substantially to mortality, morbidity, and healthcare costs, with a disproportionate impact in low- and middle-income countries like India. In India, HF prevalence is estimated at 1.3 to 4.6 million, primarily driven by ischemic heart disease. ^2, 3^ Registry data indicate that HF in India presents roughly a decade earlier than in the United States and is associated with higher in-hospital mortality, suggesting both earlier onset and more severe disease. ^4^ The HF burden is projected to increase due to rising cardiovascular risk factors and an aging population. ^5^

Heart failure with reduced ejection fraction (HFrEF) accounts for 50–60% of all HF cases and is linked to high morbidity, mortality, and healthcare utilization. ^6^ Guideline-directed medical therapy (GDMT), comprising angiotensin converting enzyme inhibitors (ACE) or angiotensin receptor blockers (ARBs) or Angiotensin receptor-neprilysin inhibitors (ARNIs), beta-blockers, mineralocorticoid receptor antagonists (MRAs), and sodium-glucose cotransporter-2 inhibitors (SGLT2i), is the cornerstone of HFrEF management, significantly improving survival and reducing hospitalizations. ^7, 8^ Indian data from the Trivandrum ^9^ and National HF Registries ^10^ demonstrate the long-term benefits of GDMT; however, uptake remains suboptimal, indicating persistent implementation gaps. ^11^

Optimizing GDMT globally could prevent an estimated 1.2 million deaths annually. ^12^ Early initiation, particularly at discharge, is strongly associated with long-term adherence and dose optimization. ^13^ However, in low- and middle-income countries like India, implementation is hindered by high out-of-pocket costs, limited drug availability, and the need for close monitoring. Additional challenges include physician inertia, limited awareness of treatment targets, poor care coordination, and inadequate follow-up systems. ^11^ Evidence from real-world studies shows that structured, protocol-driven titration improves left ventricular ejection fraction, NYHA class, and clinical outcomes. ^14^ Addressing barriers at the health system, provider, and patient levels through multi-level interventions is critical to enhancing GDMT uptake and improving outcomes in HFrEF. ^15^

Collaborative care models involving multidisciplinary teams, particularly when integrated with mobile health (mHealth) technologies, offer a promising approach to enhance GDMT implementation in resource-limited settings. Digital tools can overcome treatment initiation inertia, improve adherence, and enable task-sharing with non-physician health workers. ^16– 18^ While prior interdisciplinary and mHealth-enabled strategies, including those evaluated in both high-income and limited Indian healthcare settings, have demonstrated improvements in self-care, medication adherence, and GDMT initiation, important gaps remain in scalability and integration into routine care pathways. ^19– 22^ Furthermore, their effectiveness within the Indian healthcare system warrants rigorous evaluation through randomized controlled trials.

The TIME-HF trial was designed to evaluate the effectiveness and scalability of a nurse-led collaborative care model (CCM) supported by mHealth tools in HF management. ^23^ While the primary outcome of the TIME-HF trial is days alive and out of hospital (DAOH) over two years, a key interim process outcome is improvement in GDMT prescription at discharge. This paper presents the baseline characteristics of enrolled patients and examines GDMT use at discharge using a composite GDMT score.

## Methods

**Ethical considerations**

The participants were informed about the study and provided with a detailed information sheet. Trained research nurse appointed by the principal investigator obtained written informed consent from all study participants. The investigation conforms with the principles outlined in the *Declaration of Helsinki.* The study was approved by the Institutional Ethics Committee of Sree Chitra Tirunal Institute for Medical Sciences and Technology (SCTIMST) and the ethics committees of all participating centers (SCT/IEC/1691/AUGUST-2021). The study protocol is registered with the Clinical Trials Registry of India (CTRI/2021/11/037797).

### Study design and participants

TIME-HF was a multicenter, parallel-group, open-label, cluster-randomised controlled trial conducted across 22 centers in India. A total of 1,507 HF patients with reduced ejection fraction (HFrEF) were enrolled. Clusters, defined as eligible HF centres in major hospitals from the national or Kerala HF registry, serving ≥80 new patients in six months, and consenting to randomisation. Clusters that met predefined eligibility criteria were randomized to either a nurse-led collaborative care model or standard care, as outlined in the published protocol. ^18^ Each site operated independently with dedicated staff for patient recruitment and follow-up, maintaining comparable clinical team structures. Eligible participants were adults (>18 years) with echocardiographically confirmed HFrEF (EF <40%) who were enrolled consecutively before hospital discharge, following written informed consent.

### Randomisation and masking

Cluster randomisation was conducted at a single time point prior to trial initiation, with eligible units allocated in a 1:1 ratio to the intervention or the usual care arm. Allocation was based on a computer-generated random sequence without blocking, restriction, or matching. Eleven units were assigned to each arm ( Figure 1). As an open-label trial, neither participants nor personnel delivering the intervention were masked to group assignment. Outcome assessors and data analysts were aware of group allocation, and no formal assessment of masking was conducted.

**Figure 1.**
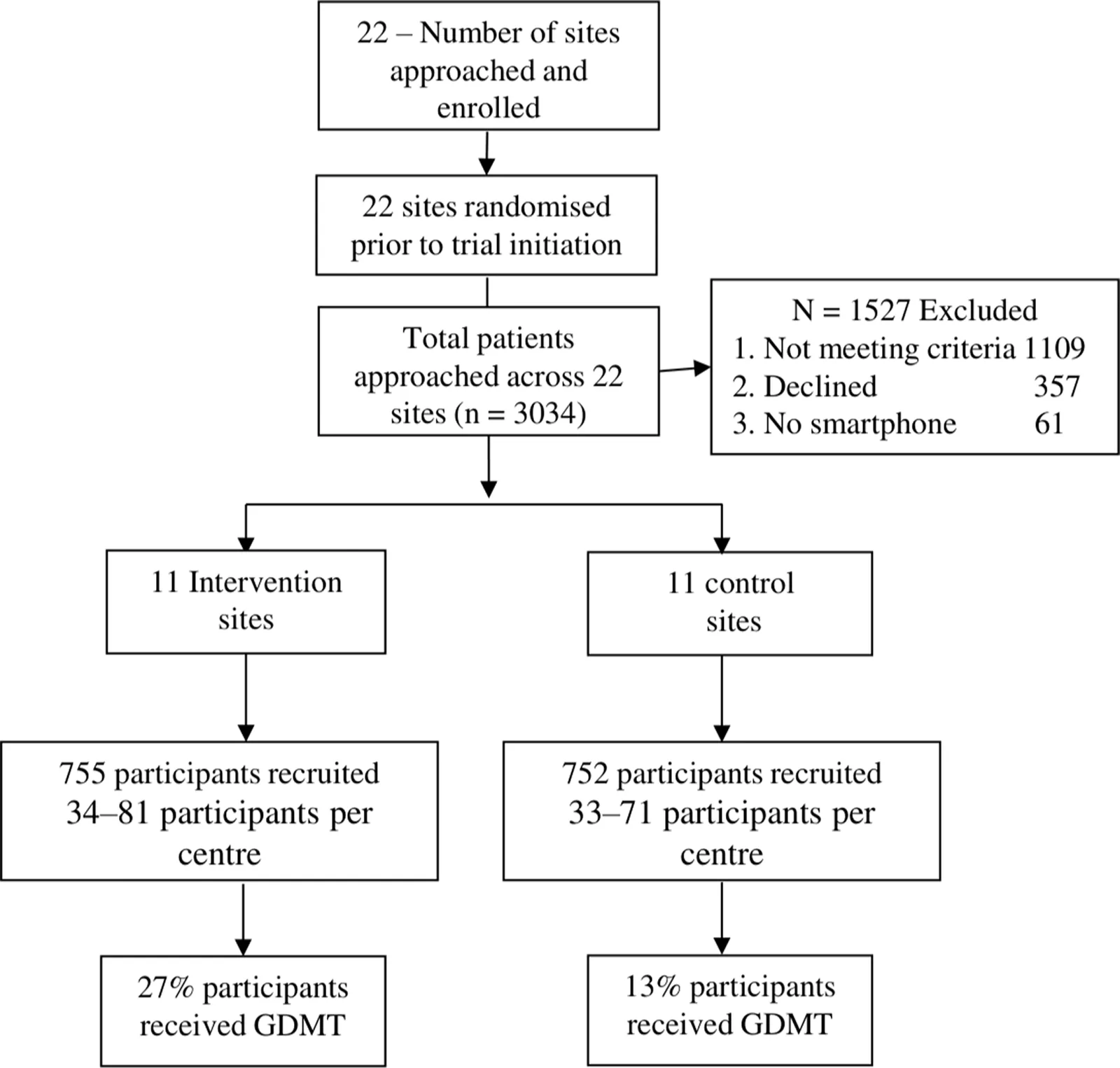
Trial flowchart.

### Procedures

Although the collaborative care model (CCM) included multiple components, this paper focuses on the interventions at the time of discharge. Patients in the usual care arm received standard care from their treating physician, with support from a clinical coordinator for data collection. In the intervention arm, care was led by trained nurses in collaboration with physicians, using existing site-specific practices to deliver integrated heart failure (HF) management. The intervention comprised three phases: (1) screening and risk stratification; (2) lifestyle modification support (e.g., nutrition, substance use, activity planning, adherence counselling); and (3) nurse-facilitated, mHealth-assisted disease management, including self-care education, follow-up, and remote monitoring.

At discharge, nurses used a customized mHealth application to review medication plans in the discharge summary and identify gaps in GDMT. The app facilitated real-time communication between patients, nurses, and physicians. Nurses could recommend initiating or modifying GDMT through the app, subject to physician approval. Once approved, updated prescriptions were communicated to patients through the mHealth platform. Although, the platform was used for post-discharge follow-up till completion of the study to reinforce GDMT recommendations and address barriers to medication use, the focus of this paper is on improvement in GDMT prescription at the time of discharge.

### Study measurements

Trained research nurses administered a structured questionnaire at baseline, prior to discharge. Data collection included HF and angina symptoms, disease etiology, comorbidities, medication use, lifestyle choices, and substance use patterns, demographic, socioeconomic, and general health information. Detailed measurement protocols are described elsewhere. ^23^

A validated GDMT score was used to evaluate both the prescription and dose optimization of guideline-directed therapies. ^24^ Renin angiotensin system inhibitors and beta-blockers were scored as 0 (not prescribed), 1 (dose <50% of target), or 2 (dose 50–100% of target). Angiotensin receptor-neprilysin inhibitors (ARNIs) were assigned a score of 3, irrespective of dose. MRAs and SGLT2 inhibitors were scored 0 if not prescribed and 2 if prescribed, regardless of dose. The composite score ranged from 0 to 9 ( Table 1). We used a pragmatic GDMT score that quantifies both prescription and dose intensity of core HFrEF therapies, which are associated with clinical outcomes and are feasible to implement in real-world practice. ^25^ Data were entered using tablet-based REDCap software by trained nurses or research coordinators under the supervision of principal investigators. The coordinating team conducted data cleaning, query resolution, and analysis. All data were de-identified to maintain confidentiality.

**Table 1.** Simple GDMT Score.

Type of drug	Dose	Simple GDMT Score
**RASi**	None	0
	<50% of maximum daily dose of ACEi/ARB	1
	≥50% of maximum daily dose of ACEi/ARB	2
	ARNI (any dose)	3
**Beta-blockers **	None	0
	<50% of maximum daily dose	1
	≥50% of maximum daily dose	2
**MRA**	None	0
	Any dose	2
**SGLT2i**	None	0
	Any dose	2
Total maximum Score = 9 * High Score ≥ 5, Low score < 5		

RASi-Renin Angiotensin System inhibitors; ACEi-Angiotensin converting enzyme inhibitors; ARB- Angiotensin Receptor Blockers; ARNI- Angiotensin Receptor Neprilysin Inhibitor; MRA-Mineralocorticoid Receptor Antagonist; SGLT2i-Sodium-Glucose cotransporter-2 inhibitors.

### Outcomes

While the primary outcome of the TIME-HF trial was days alive and out of hospital (DAOH) over two years, a key interim process outcome was the improvement in GDMT prescription at discharge. This was measured using a validated score that captured both initiation and dose optimization of GDMT ( Table 1). As an indicator of intervention fidelity, this outcome provided early insight into the collaborative care model's potential to enhance long-term clinical outcomes by enabling the timely implementation of evidence-based therapy. The GDMT score ranged from 0 to 9, with scores of 5 or higher categorized as ‘high’ and those below 5 as ‘low’. As this paper examines GDMT prescription at discharge, it assesses the one-time nurse-led intervention delivered immediately before discharge, which involved reviewing medications in the discharge summary using an mHealth application. The application flagged missing GDMT components and generated an alert for the nurse, who then consulted the physician, initiated the treatment, or documented the reasons for non-prescription.

### Sample size and power

The primary outcome of our trial was days alive and out of hospital (DAOH). Our sample size calculation was based on the two-year DAOH data reported in the Trivandrum Heart Failure Registry. ^9^ We anticipated an average DAOH (Days Alive and Out of Hospital) of approximately 566 days in the control group, with a standard deviation of 69 days at two years. We projected that the intervention would increase DAOH by 3% (586 days in the intervention group). To detect this difference with 91% power at a two-sided 5% significance level, we calculated that 770 participants per arm (1,540 total) are needed. These participants are organized into 11 clusters per group (22 clusters overall), assuming an ICC of 0.01 (design effect of 2.08) and a coefficient of variation of cluster size of 0.85. The outcome of interest in the current paper was the GDMT rate at discharge. Since precise data on the prevalence of the four pillars of GDMT were not available, the sample size ensured more than 80% power to detect a clinically meaningful 7–10% increase in the GDMT rate, assuming a baseline prevalence of 5-50% and an intracluster correlation coefficient (ICC) between 0.01 and 0.05 ( Figure 2). In the majority of scenarios, the study achieved a power exceeding 80%.

**Figure 2.**
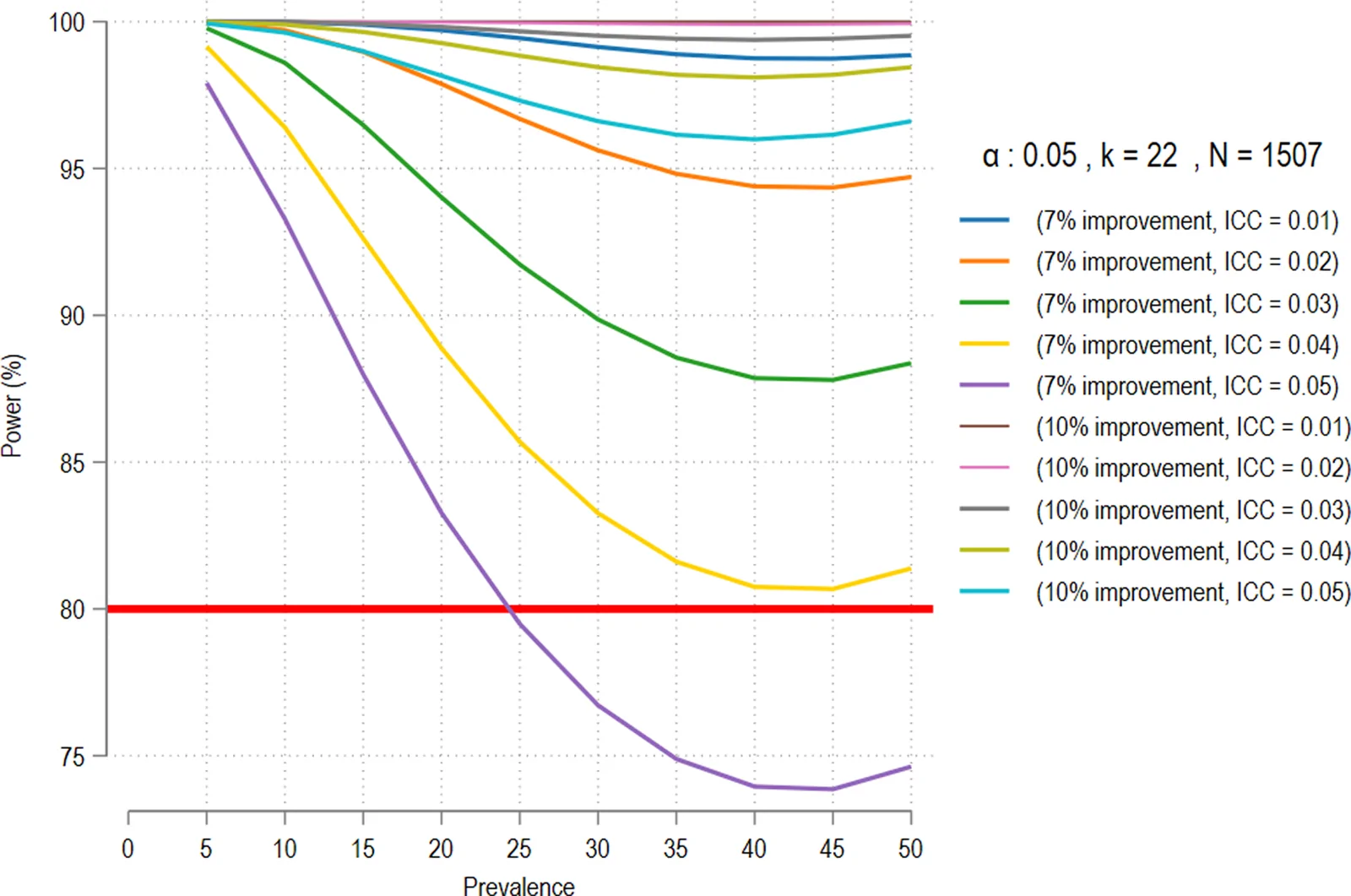
Estimated statistical power function based on GDMT four-pillar prevalence assumptions. *N = sample size; α = significance level; K = number of clusters; ICC = Intraclass Correlation Coefficient.

### Statistical analysis

Quantitative analyses were conducted in accordance with CONSORT guidelines for cluster-randomized trials. ^26^ Continuous variables were summarized using means and standard deviations, and categorical variables using frequencies and percentages. Baseline characteristics were compared across treatment groups. The effect of the intervention on GDMT prescription rates was assessed using a multivariable mixed-effects logistic regression model accounting for random effects at both the cluster and district levels. The model was adjusted for age, education, body mass index (BMI), left ventricular ejection fraction (LVEF), ischemic heart disease (IHD), tobacco use, alcohol consumption, hypertension (HTN), diabetes mellitus (DM), atrial arrhythmia, cerebrovascular accident (CVA), chronic obstructive pulmonary disease (COPD), and chronic kidney disease (CKD). All analyses were performed using Stata version 16.0 (StataCorp, College Station, TX, USA).

### Role of the funding source

This study was supported by the Wellcome Trust/DBT India Alliance through a Clinical and Public Health Intermediate Fellowship awarded to PJ [IA/CPHS/20/1/505229]. The funding agency had no role in the study’s design, implementation, data collection, analysis, interpretation, or reporting. They were not involved in the preparation of this manuscript. The authors are solely responsible for the content and writing of the paper.

## Results

### Baseline

Participants were recruited between September 29, 2022, and September 30, 2023, from eligible units participating in the trial. The study enrolled 1,507 heart failure patients, with 755 randomized to the intervention group and 752 to the control group.

Socio-demographic Profile:

The mean age of the study population was 61.9 years (SD = 12.1), with males comprising 77.6% (n = 1,169) of participants. More than half (57.3%, n = 863) resided in rural areas, and 37.2% (n = 561) were classified as living below the poverty line. The median educational attainment was up to the 10 ^th^ standard, with an interquartile range of 7 to 12 years of formal education ( Table 2).

Risk Factors and Comorbidities:

**Table 2.** Baseline socio-demographic and clinical characteristics of study population.

Characteristic	Overall, N = 1507	Intervention, N = 755	Control, N = 752
**Socio-demographic **			
Age	61.9 (12.1) ^*^	62.4 (12.1) ^*^	61.4 (12.2) ^*^
Male gender	1169 (77.6%)	585 (77.5%)	584 (77.7%)
Years of education	10 (7,12) ^†^	10 (8,12) ^†^	10 (5,11) ^†^
Rural area of residence	863 (57.3%)	397 (52.6%)	466 (62.0%)
BPL ration card holders	561 (37.2%)	195 (25.8%)	366 (48.7%)
**Risk factors**			
Tobacco use (past or current)	409 (27.1%)	162 (21.5%)	247 (32.8%)
Alcohol use (past or current)	393 (26.1%)	146 (19.3%)	247 (32.8%)
**Co-morbidities **			
Diabetes mellitus	935(62%)	512 (67.8%)	423 (56.3%)
Hypertension	846 (56.1%)	457 (60.5%)	389 (51.7%)
Chronic Kidney Disease	278 (18.4%)	131 (17.4%)	147 (19.5%)
COPD/Asthma	160 (10.6%)	69 (9.1%)	91 (12.1%)
Hypothyroidism	120 (8%)	79 (10.5%)	41 (5.5%)
Stroke/TIA	97(6.4%)	56 (7.4%)	41 (5.5%)
Atrial arrhythmia	72 (4.8%)	41 (5.4%)	31 (4.1%)
Hyperthyroidism	9 (0.6%)	4 (0.5%)	5 (0.7%)
**Aetiology of heart failure**			
Ischemic Heart Disease	1166 (77.4%)	568 (75.2%)	598 (79.5%)
Dilated cardio myopathy	406 (26.9%)	216 (28.6%)	190 (25.3%)
Non rheumatic valvular heart disease	68 (4.5%)	6 (0.8%)	62 (8.2%)
Right heart failure	43 (2.9%)	35 (4.6%)	8 (1.1%)
Rheumatic heart Disease	34 (2.3%)	15 (2%)	19 (2.5%)
Congenital heart disease	9 (0.6%)	7 (0.9%)	2 (0.3%)
Peripartum cardio myopathy	6 (0.4%)	5 (0.7%)	1 (0.1%)
Myocarditis	4 (0.3%)	2 (0.3%)	2 (0.3%)
Hypertrophic cardio myopathy	3 (0.2%)	3 (0.4%)	0 (0%)
Restrictive cardio myopathy	2 (0.1%)	0 (0%)	2 (0.3%)
Infective endocarditis	2 (0.1%)	2 (0.3%)	0 (0%)
**Clinical characteristics**			
NYHA class III/IV	707 (46.9%)	409 (54.1%)	298 (39.6%)
Heart rate	83.4 (16.9) ^*^	82 (16.9) ^*^	84.8 (16.7) ^*^
Systolic blood pressure	125.2 (22.3) ^*^	126.3 (22.3)	124.2 (22.3)
Diastolic blood pressure	77.7 (12.8) ^*^	78.4 (12.5) ^*^	76.9(13.1) ^*^
Ejection fraction	31.1 (5.9) ^*^	31.4 (6.1) ^*^	30.7 (5.9) ^*^
Serum Creatinine	1.2 (0.9,1.6) ^†^	1.2 (1,1.5) ^†^	1.2 (0.9,1.7) ^†^
Serum sodium	136 (134,139) ^†^	136 (134,139) ^†^	137 (134,139) ^†^
Serum Potassium	4 (3.7,4.4) ^†^	4 (3.6,4.3) ^†^	4.1 (3.8,4.5) ^†^

BPL-Below Poverty Line, TIA-Transient Ischemic Attack, COPD-Chronic Obstructive Pulmonary Disease, NYHA-New York Heart Association.

* mean (standard deviation);

^†^ median (25%, 75%); n (%).

Among the study participants, 27.1% (n = 409) reported current or past tobacco use, and 26.1% (n = 393) reported current or previous alcohol consumption. Comorbid conditions were common: 56.1% (n = 846) had hypertension, and 62.0% (n = 935) had diabetes mellitus. Atrial arrhythmias were observed in 4.8% (n = 72) of participants. A history of stroke or transient ischemic attack (TIA) was documented in 6.4% (n = 97). Hypothyroidism and hyperthyroidism were reported in 8% (n = 120) and 0.6% (n = 9) of participants, respectively. Respiratory conditions such as COPD or asthma were reported in 10.6% (n = 160). Chronic kidney disease (CKD) was present in 18.4% (n = 278) of participants, of whom 10 participants (0.7%) were receiving end-stage renal replacement therapy ( Table 2).

Aetiology of Heart Failure:

Ischemic heart disease was the leading cause of heart failure, observed in 77.4% of patients (n = 1,166). Dilated cardiomyopathy was the second most common etiology, affecting 26.9% (n = 406). Less frequent causes included non-rheumatic valvular heart disease (4.5%, n = 68), right heart failure (2.9%, n = 43), and rheumatic heart disease (2.3%, n = 34). Other rare etiologies, such as congenital heart disease, peripartum cardiomyopathy, myocarditis, and infective endocarditis, were documented in a small number of cases ( Table 2).

Other Clinical Characteristics:

Nearly half of the cohort (46.9%) were classified as NYHA class III or IV, indicating moderate to severe functional limitation. The mean heart rate was 83.4 bpm (SD = 16.9), with an average blood pressure of 125.2/77.7 mmHg (SD = 22.3/12.8). Echocardiography showed a mean ejection fraction of 31.1% (SD = 5.9), confirming HFrEF. Median serum creatinine was 1.2 mg/dL (IQR: 0.9–1.6). Baseline sociodemographic and clinical characteristics were comparable between intervention and control groups ( Table 2).

### Guideline-Directed Medical Therapy (GDMT) prescription patterns

Among all patients, 50% received RAS inhibitors, 32.5% received triple therapy (RASi, beta-blockers, MRAs), and 20.1% were prescribed all four GDMT components, including SGLT2 inhibitors. Prescription rates were higher in the intervention group: 55.8% for RASi, 36.6% for triple therapy, and 27.3% for all four components, compared to 44.3%, 28.5%, and 13% in the control group, respectively ( Figure 3).

**Figure 3.**
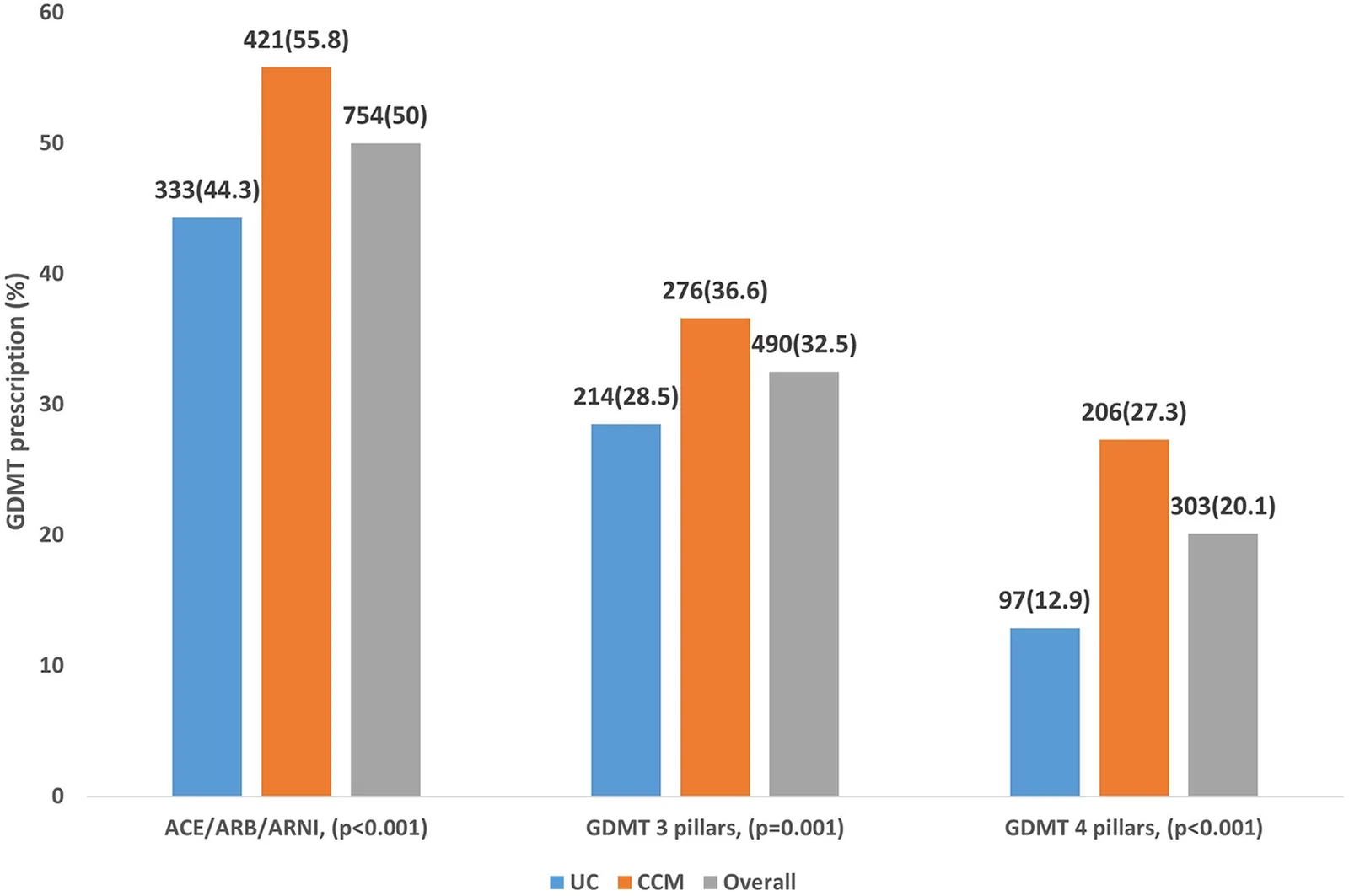
GDMT prescription rate at discharge. RASi-Renin Angiotensin System inhibitors; ACEi-Angiotensin converting enzyme inhibitors; ARB- Angiotensin Receptor Blockers; ARNI- Angiotensin Receptor Neprilysin Inhibitor; Beta-blockers: Beta-adrenergic blockers; MRA-Mineralocorticoid Receptor Antagonist; SGLT2i-Sodium-Glucose cotransporter-2 inhibitors, UC: Usual care (Control), CCM: collaborative care model (Intervention), Values are expressed as n (%). The p values indicate comparison between usual care group and intervention group.

The proportion of patients receiving all four pillars of GDMT was significantly higher in the intervention group (27.3%) than in the control group (13%) [p < 0.001; adjusted OR = 1.61, 95% CI: 1.48–1.75] ( Figures 3 and 4). More than 50% of the maximum daily dose of beta blockers and RASi was received by 10.7% and 5.6% of the study population, respectively ( Table 3). A low GDMT score (<5) was reported by 61.8% in the usual care group and 52.1% in the intervention group ( Figure 5). The intervention group participants were 13% more likely to achieve a ‘high GDMT score’ than the usual care group participants (OR = 1.13, 95% CI: 1.04-1.22, p = 0.003) ( Figure 4). The intra-class correlation coefficient (ICC) analysis showed that participants within the same cluster did not exhibit greater similarity in GDMT use than those across clusters. While variation in the prescription of the four pillars of GDMT was negligible across clusters (ICC = 0.00022; 95% CI: 0.00008-0.00059), it was pronounced across districts (ICC = 0.3806; 95% CI: 0.2615-0.5161) ( Figure 4).

**Figure 4.**
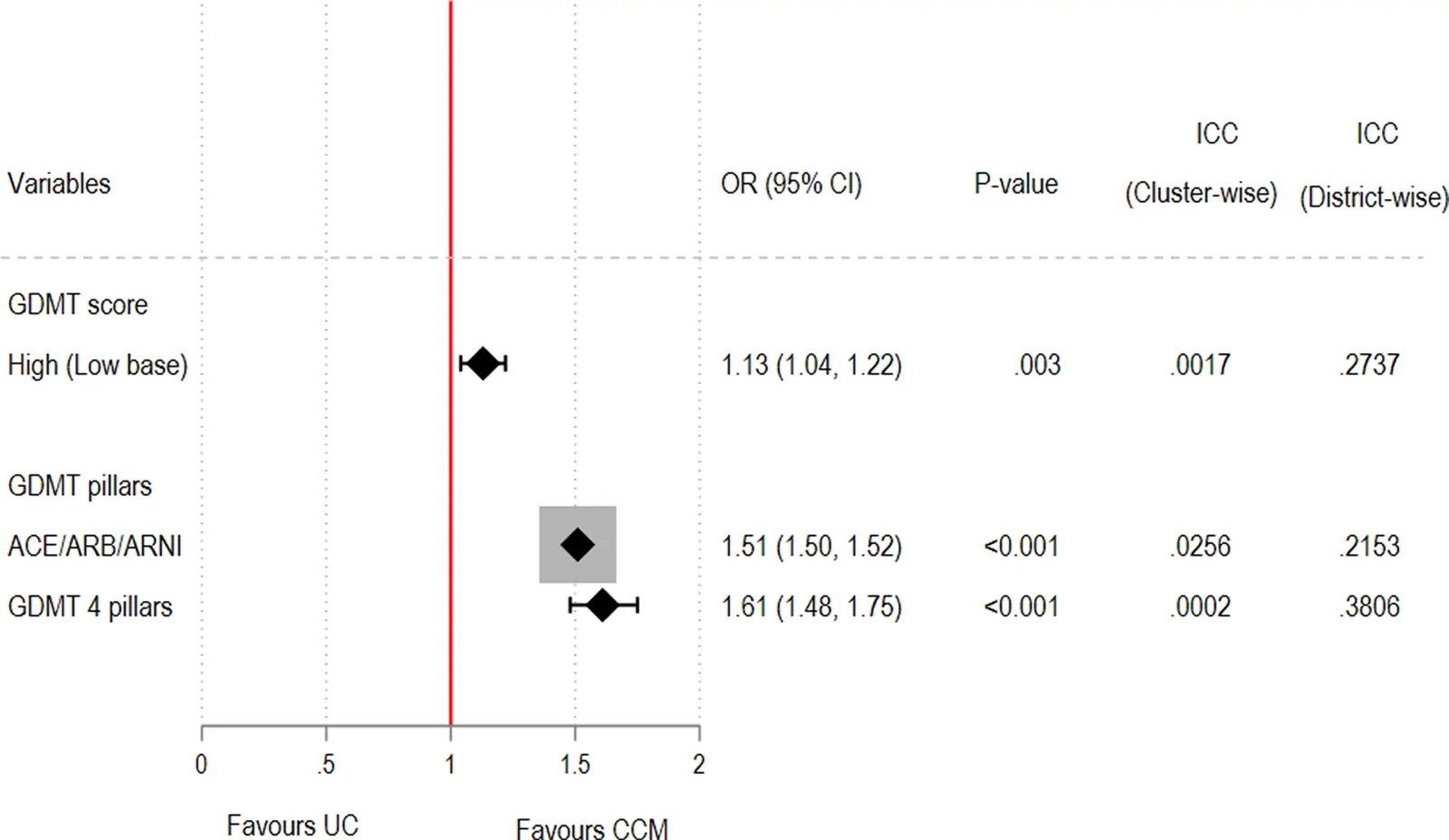
Regression model with odds ratio and 95% confidence interval. RASi-Renin Angiotensin System inhibitors; ACEi-Angiotensin converting enzyme inhibitors; ARB- Angiotensin Receptor Blockers; ARNI- Angiotensin Receptor Neprilysin Inhibitor; Beta-blockers: Beta-adrenergic blockers; MRA-Mineralocorticoid Receptor Antagonist; SGLT2i-Sodium-Glucose cotransporter-2 inhibitors: Usual care (Control), CCM: collaborative care model (Intervention): ICC = Intraclass Correlation Coefficient.

**Table 3.** GDMT score in the study population.

Type of drug	Dose	Simple GDMT Score	Intervention N (%)	Control N (%)	Total N (%)
RASi	None	0	334(44.2)	419(55.7)	753(50.0)
	<50% of maximum daily dose of ACEi/ARB	1	216(28.6)	175(23.3)	391(30.0)
	≥50% of maximum daily dose of ACEi/ARB	2	45(6.0)	39(5.2)	84(5.6)
	ARNI (any dose)	3	160(21.2)	119(15.8)	279(18.5)
Beta- blockers	None	0	205(27.2)	223(29.7)	428(28.4)
	<50% of maximum daily dose	1	456(60.4)	461(61.3)	917(60.8)
	≥50% of maximum daily dose	2	94(12.5)	68(9)	162(10.7)
MRA	None	0	268(35.5)	181(24.1)	449(29.8)
	Any dose	2	487(64.5)	571(75.9)	1058(70.2)
SGLT2i	None	0	350(46.4)	420(55.9)	770(51.1)
	Any dose	2	405(53.6)	332(44.1)	737(48.9)

RASi-Renin Angiotensin System inhibitors; ACEi-Angiotensin converting enzyme inhibitors; ARB- Angiotensin Receptor Blockers; ARNI- Angiotensin Receptor Neprilysin Inhibitor; MRA-Mineralocorticoid Receptor Antagonist; SGLT2i- Sodium-Glucose cotransporter-2 inhibitors.

**Figure 5.**
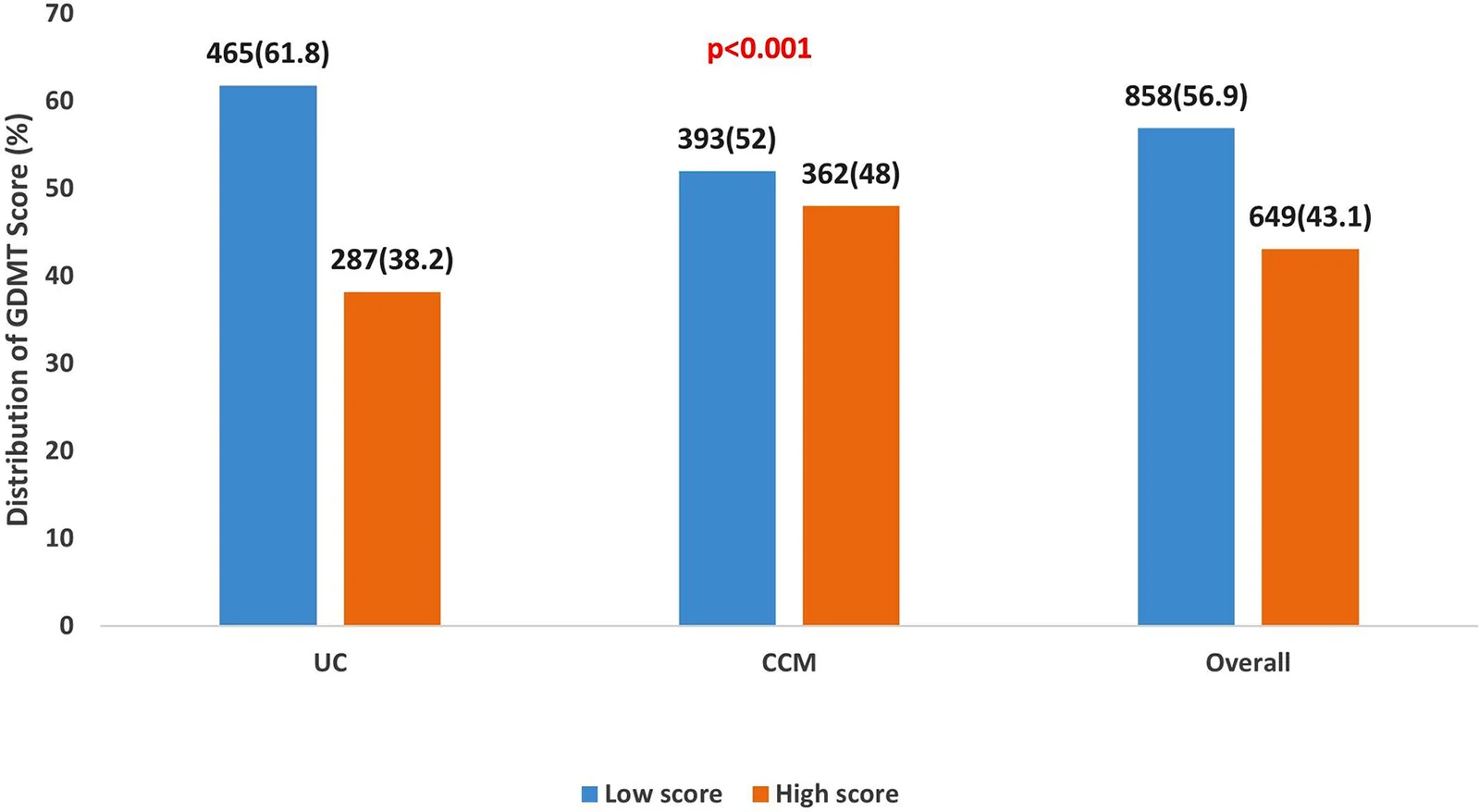
Simple Guideline Directed Medical Therapy (GDMT) Score. * High score: GDMT score ≥ 5, low score: GDMT score <5, UC: Usual care (Control), CCM: collaborative care model (Intervention), Values are expressed as n (%). The p value indicates comparison between usual care group and intervention group.

## Discussion

The TIME-HF study showed that a nurse-led, mHealth-enabled collaborative care model significantly improved GDMT prescription at discharge in patients with HFrEF. Despite overall low GDMT uptake, the intervention group had substantially higher rates of RAS inhibitor use across all four GDMT components compared with usual care. The likelihood of receiving all four pillars of GDMT was more than 60% higher in the intervention group, highlighting the importance of structured discharge reviews in promoting evidence-based HF management in resource-limited settings.

Our study found that patients in the intervention group had significantly higher discharge GDMT scores than those in the control group. These results are consistent with a 2024 quality improvement initiative, which demonstrated that structured, nurse practitioner and pharmacist-led programs can effectively achieve target GDMT doses despite known implementation barriers. ^27^ Our findings support the premise that structured discharge planning, when led by trained non-physician providers and supported by mHealth tools, can help bridge critical care gaps. This aligns with prior evidence from the COACH and other trials, which highlighted the benefits of nurse-led disease management programs in high-income settings. ^28, 29^ The observed improvement further reinforces the 2024 ACC Expert Consensus Statement, which advocates for multidisciplinary team-based approaches to optimize GDMT uptake in HF care. ^30^ A 2023 systematic review and meta-analysis by Zheng et al. showed that such team-based approaches significantly improved the initiation and titration of renin-angiotensin system inhibitors (RASi) and beta-blockers. ^31^ Notably, the increased use of ARNIs in the intervention group (21.5% vs. 15.8%) is particularly significant considering the 2022 AHA/ACC/HFSA Guidelines, which strongly recommend ARNIs as a foundational therapy for HFrEF. ^32^ However, the overall modest prescription rate of ARNIs highlights ongoing challenges, including high costs, the need for close monitoring, and clinical inertia. ^33^ These barriers reflect a broader pattern of underutilization of evidence-based therapies and underscore the importance of structured nurse-led interventions, patient education, and systematic follow-up to improve implementation and overcome treatment inertia.

Despite the intervention’s positive impact, overall GDMT prescription rates and dose optimization remained suboptimal in our study. This reflects broader national and global trends, with persistent gaps in heart failure management. Physician-only care models often delay GDMT initiation and titration, as illustrated by the CHAMP-HF registry, in which only 1.1% of patients received all three foundational therapies at ≥50% target doses. ^34^ Similarly, the PINNACLE India Program reported low GDMT uptake despite physician-focused interventions. ^35^ Low rates of dose optimization are consistent with prior studies showing that clinical instability (including hypotension, renal dysfunction, and congestion), polypharmacy, fraility, and limited early post-discharge follow-up can constrain rapid titration of GDMT, even in structured intervention programs such as STRONG-HF. ^36^ In contrast, our nurse-led, task-sharing model demonstrated greater feasibility and scalability within resource-limited LMIC settings, highlighting the effectiveness of collaborative care in advancing guideline-based HF treatment.

Mobile health technologies offer a promising means to engage cardiac patients in self-management while strengthening existing clinical care pathways, ^37^ with recent Indian evidence from GOALS-HF demonstrating that remote monitoring–supported models can facilitate GDMT optimization. ^38^ This approach aligns with global and national strategies, such as the WHO's HEARTS technical package and India’s Ayushman Bharat Digital Health Mission, which promote community-level, digitally supported chronic disease management. ^39^ The TIME-HF study extends this evidence to HF care in India, demonstrating that task-shifting to trained non-physician providers, when integrated with mHealth tools, is both feasible and effective.

Our findings provide a scalable model for enhancing HF care across healthcare settings in India.

The study population was predominantly older males, most from rural areas, and over one-third lived below the poverty line. These demographics align with findings from national HF registries in India, which highlight a higher HF burden among socioeconomically disadvantaged and rural populations. ^40, 41^ The high rates of diabetes (62%) and hypertension (56.1%) in our study reflect India's ongoing shift toward non-communicable disease-driven cardiovascular morbidity. ^5^ These findings underscore the critical need to improve HF care in India, especially given the high burden of comorbidities, socioeconomic disadvantage, and limited access to specialized cardiac services.

A major strength of this study is its cluster-randomized design and pragmatic implementation across diverse healthcare settings in India, which enhances the external validity and generalizability of the findings. The TIME-HF trial represents one of the most extensive contemporary evaluations of patients with HFrEF in the Indian context, providing valuable insights into real-world prescribing practices. The use of a validated GDMT score that incorporates both drug class and dosing allow for a nuanced assessment of prescription quality, rather than simply evaluating drug initiation. As a discharge-level prescribing measure, GDMT use is primarily driven by individual patient characteristics, contraindications, and clinical stability, which may explain the low cluster-level ICC observed, whereas the substantial district-level clustering suggests an essential influence of broader regional and health-system factors on GDMT implementation; accordingly, cluster randomization was chosen to minimize contamination and to evaluate downstream outcomes such as days alive and out of hospital (DAOH) over longitudinal follow-up. ^42^

Our study highlights the feasibility and impact of nurse-led, mHealth-enabled collaborative care models in improving evidence-based therapy at discharge. However, several limitations should be considered. This interim analysis focuses solely on discharge prescription patterns. It does not evaluate long-term clinical outcomes, such as days alive and out of hospital, mortality, hospital readmissions, or quality of life, which are critical for assessing the sustained impact of GDMT optimization. Additionally, the study did not evaluate medication adherence post-discharge, did not directly address medication affordability or long-term access, did not include patient-reported outcomes, and did not systematically capture adverse events, intolerance, or specific reasons for non-initiation of GDMT, which are essential to understanding the real-world effectiveness of the intervention. The absence of these measures limits the ability to capture patient engagement and long-term benefit fully. These will be addressed in future analyses once the full two-year follow-up is complete. It will incorporate a broader range of outcomes, including adherence metrics and patient-centered endpoints, to provide a more comprehensive evaluation of collaborative care strategies in heart failure management.

## Conclusion

In conclusion, the TIME-HF study shows that a structured, nurse-led intervention substantially increased the prescription of GDMT at discharge, with rates exceeding 60% in the intervention group compared with usual care. These findings underscore the effectiveness of collaborative care models, particularly those integrating trained nurses and mHealth tools, in strengthening evidence-based HF management in resource-limited settings. The study provides critical insights into the current landscape of HF care in India and offers a scalable framework for optimizing GDMT implementation.

## Data Availability

The data that supports the findings of this study can be accessed from the repository as follows. Figshare: TIME HF GDMT data. https://figshare.com/articles/dataset/TIME_HF_GDMT_data/31898050?file=63664761. ^43^ Figshare: Structured Questionnaire-TIME HF GDMT data. https://doi.org/10.6084/m9.figshare.31948923. ^44^ Figshare: Informed Consent (TIME-HF study). https://doi.org/10.6084/m9.figshare.22360375. ^45^ The file “Informed Consent (TIME-HF study)” contains the following extended data
• Participant information sheet and • Consent form Figshare. CONSORT TIME HF GDMT. https://doi.org/10.6084/m9.figshare.31914033. ^46^ The file CONSORT TIME HF GDMT contains the following extended data
• CONSORT checklist • CONSORT diagram Data are available under the terms of the Creative Commons Attribution 4.0 International license (CC-BY 4.0).
