# Supplementary material for: Enhancing Guideline-Directed Medical Therapy at Discharge Through Collaborative Care: Evidence from the TIME-HF Cluster Randomized Controlled Trial
Source: Wellcome Open Res. 2026 May 20;11:293. [Version 1] doi: 10.12688/wellcomeopenres.26369.1 (PMC13650326; doi:10.12688/wellcomeopenres.26369.1)
Supplement: Peer review history

# Reviewer report: Reviewer response for version 1

**DOI:** 10.21956/wellcomeopenres.29040.r160153

**Published:** 2026-08-31

**Authors:** Ambalam M Chandrasekaran

**Affiliations:** 1 Centre for Chronic Disease Control, New Delhi, Delhi, India

I congratulate the team for this much needed work to address the poor adoption of GDMT and long term collaborative care with a nurse led  health care model. Such models were rarely tested in settings like India with a very high physician dependent care. 

 Some of the observations

 1. The method mentioned recruitment of consecutive participants. The trial flowchart mentioned nearly 1109 / 3034 screened participants without describing the reasons. The reasons may be expanded to give clarity. 

 2. Has there been instances of patients who were randomized and died before discharge? If yes, this could be included in the numbers randomized and marked as unevaluable as they didn't survive and excluded from the study.

 3. There is substantial differences in the years of education (IQR 8,12 versus 5,11), rural area of residence, BPL ration card holders, risk factors and and co-morbidities. Also, data on hospital characteristics is missing. Preferably, a sub-group analysis with these key Social-economic factors (participant level) and also, centre characteristics (public versus private; teaching versus non-teaching) can be presented for GDMT rates.

 4. Study settings - The mobile phone penetration is substantial as evident by the exclusion because of no smartphone is negligible in the study centres. The work is predominantly done in southern India with a high-literacy rate. These can be considered asset for the clinical trial settings, but raises concerns over the validity of findings in other settings within India itself.

 Once again, I congratulate the study team on this important work, which will be a landmark point in the nurse-led HF clinics and improved adoption of GDMT drugs.

Is the work clearly and accurately presented and does it cite the current literature?

Yes

If applicable, is the statistical analysis and its interpretation appropriate?

Yes

Are all the source data underlying the results available to ensure full reproducibility?

Yes

Is the study design appropriate and is the work technically sound?

Yes

Are the conclusions drawn adequately supported by the results?

Yes

Are sufficient details of methods and analysis provided to allow replication by others?

Partly

Reviewer Expertise:

Clinical trials, cardiac rehabilitation, yoga, lifestyle medicine

I confirm that I have read this submission and believe that I have an appropriate level of expertise to confirm that it is of an acceptable scientific standard, however I have significant reservations, as outlined above.

# Response

**Published:** 2026-09-03

**Authors:** Panniyammakal Jeemon

**Affiliations:** Sree Chitra Tirunal Institute for Medical Sciences and Technology, Trivandrum, India

Thank you very much for the comments.   

 As specified in the study protocol, consecutive patients were screened at the participating centres and assessed against the predefined eligibility criteria. The primary eligibility criterion was a standard diagnosis of heart failure with reduced ejection fraction (LVEF <40%) based on echocardiography. Of the 3,034 patients screened, 1,109 were excluded for not meeting the predefined eligibility criteria, 357 declined participation, and 61 were excluded due to the absence of a smartphone. These reasons for exclusion are presented in the published participant flow diagram.  

 We would like to clarify that participants were recruited shortly before discharge and, therefore, patients who died before discharge did not meet the study inclusion criteria and were not recruited into the study. Individual patients were not randomized in this trial. As specified in the study protocol, eligible units were randomized at one time point, prior to trial implementation, in a 1:1 ratio using computer-generated random numbers. Consecutive eligible patients were subsequently recruited from the participating centres. Therefore, there were no randomized participants who died before discharge and were subsequently excluded from the study on account of death. Participant outcomes, including deaths occurring during follow-up, were accounted for according to the prespecified study procedures.

   Given the cluster-randomized design, we do not have sufficient statistical power for meaningful subgroup analyses based on these participant- and centre-level characteristics. Such analyses would therefore be exploratory and potentially underpowered.   We acknowledge that the predominantly southern Indian setting may have relatively higher digital penetration. However, the Government of India’s Comprehensive Modular Survey: Telecom (2025) reported that 85.5% of households nationally owned a smartphone and 86.3% had internet access at home ( Press Information Bureau). Although digital access is increasingly widespread, regional differences in literacy, connectivity and healthcare infrastructure may affect implementation; therefore, generalizability to substantially different settings should be interpreted cautiously.

# Reviewer report: Reviewer response for version 1

**DOI:** 10.21956/wellcomeopenres.29040.r156156

**Published:** 2026-06-16

**Authors:** Jaideep C Menon

**Affiliations:** 1 Amrita Vishwa Vidyapeetham, Kochi, Kerala, India

I read with considerable interest the manuscript titled ‘Enhancing Guideline-Directed Medical Therapy at Discharge Through Collaborative Care: Evidence from the TIME-HF Cluster Randomized Controlled Trial’, authored by Jeemon P and colleagues.

 I have the following observations and comments to make and seek some clarification as well- 

 Since the current manuscript looks at discharge prescriptions in patients with HFrEF is it logical to give a scale based on 50% and 100% of maximum dose, given that usual up titration is done within 1-2 weeks and not usually in the index admission.

 50% and 100% are based on the maximum dose of the drug I presume and not on target blood pressure cut-offs. Wouldn’t the BP have been a better surrogate, given that the mean LVEF in both control and intervention arms was 30%.

 How many of the participants were already on treatment for HF from the cohort of patients in both arms.

 Was a uniform protocol used to train the nurses in data entry and was a refresher training done for the same. How was training of nurses done and what was the protocol in case the trained nurse quit the facility

 Was there a difference in GDMT between facilities – Public institutions versus private, given that the majority of the institutions were from the corporate, private sectors.

 There is a significant difference in numbers of BPL card holders, tobacco and alcohol users in the control arm; a 10% difference between intervention and control for diabetes and hypertension. Could this have confounded results vis a vis ACE/ARB/ARNI and SGLT 2 inhibitors.

 Would a App based prompt have helped in guiding the discharge prescription given that nurses tend to have a high turnover in most settings.

 I congratulate the researchers on their effort and am sure that the TIM-HF will help direct therapy for heart failure through this model and others.

Is the work clearly and accurately presented and does it cite the current literature?

Yes

If applicable, is the statistical analysis and its interpretation appropriate?

I cannot comment. A qualified statistician is required.

Are all the source data underlying the results available to ensure full reproducibility?

No source data required

Is the study design appropriate and is the work technically sound?

Yes

Are the conclusions drawn adequately supported by the results?

Yes

Are sufficient details of methods and analysis provided to allow replication by others?

Yes

Reviewer Expertise:

Cardiovascular disease epidemiology; population health sciences; venomous snakebite

I confirm that I have read this submission and believe that I have an appropriate level of expertise to confirm that it is of an acceptable scientific standard.

# Response

**Published:** 2026-09-03

**Authors:** Panniyammakal Jeemon

**Affiliations:** Sree Chitra Tirunal Institute for Medical Sciences and Technology, Trivandrum, India

Thank you very much for the comments. 

 We agree that achieving maximum doses during the index admission is not necessarily the objective of inpatient management. Our dose-based score was intended to quantify the intensity of GDMT prescribed at discharge rather than achievement of the maximum tolerated dose.

 We agree that blood pressure is an important consideration in GDMT initiation and titration; however, as our objective was to characterise the intensity of GDMT prescription at discharge, we retained the dose-based approach. Baseline information on whether participants were already receiving HF-directed treatment prior to the index admission was not collected as part of the study. Therefore, we were unable to determine the proportion of participants in each arm who were already receiving HF treatment before admission. Nurses involved in the intervention received standardised training on HF management, study procedures, data collection and use of the mHealth application before commencing their study responsibilities. Training was supported by nurse handbooks and the decision-support system within the mHealth application. Refresher training was provided during the study period to reinforce the study procedures. The same training procedures were followed when new nurses joined or replaced trained nurses, ensuring continuity and standardisation of the intervention and data collection processes.

 The trial was primarily designed to evaluate the effect of the cluster-randomised collaborative care intervention and was not specifically powered to compare GDMT prescribing between government and private facilities. Since this was a cluster randomized trial with only 22 centers, it is possible for some baseline differences between the treatment groups. These variables were considered in the adjusted model, and the intervention effect remained consistent after adjustment. In TIME-HF, patients not receiving any GDMT pillar were flagged by the mHealth application, which alerted the nurse for review. The nurse then communicated with the treating physician regarding initiation of appropriate GDMT, with the final prescription determined by the physician.
